# World Allergy Organization Study on Aerobiology for Creating First Pollen and Mold Calendar With Clinical Significance in Islamabad, Pakistan; *A Project of World Allergy Organization and Pakistan Allergy, Asthma & Clinical Immunology Centre of Islamabad*

**DOI:** 10.1097/WOX.0b013e31826421c8

**Published:** 2012-09-15

**Authors:** Shahid Abbas, Connie H Katelaris, Anand B Singh, Syed M Raza, Mir Ajab Khan, Muhammad Rashid, Maryam Abbas, Muhammad Ismail

**Affiliations:** 1 Allergy and Asthma Centre, Khyber Plaza, Blue, Area, Islamabad, Pakistan; 2 University of Western Sydney, Campbell town, New South Wales, Australia; 3 Institute of Genomics and Integrative Biology, Allergy and Aerobiology Laboratory, Delhi, India; 4 Rawalpindi Medical College, Islamabad, Pakistan; 5 Department of Plant Sciences, Quaid e Azam University, Islamabad, Pakistan; 6 Allergy & Asthma Centre, Allergy & Clinical Immunology, Islamabad, Pakistan; 7 Lahore Medical and Dental College, Lahore, Pakistan; 8 Institute of Biomedical and Genetic Engineering, Islamabad, Pakistan

**Keywords:** pollen and mold calendar, aerobiology, Islamabad, Burkard spore trap, *Broussonetia papyrifera*, *Cannabis sativa*, skin prick test, SPT, asthma, allergic rhinitis

## Abstract

Pollen and mold allergies are highly problematic in Islamabad. This study was conducted to investigate the type and concentration of airborne pollens/molds causing allergic diseases in susceptible individuals. A volumetric spore trap (Burkard) was placed at the height of 11 m and ran continuously for 3 years. Once a week, the collecting drum was prepared by affixing Melinex tape with a double sided adhesive that was coated with a thin layer of silicone grease. Every Sunday at 9:00 AM the drum was replaced by another drum and the pollen/mold spores were removed and permanently mounted on slides. Using a microscope, the trapped particles were identified and recorded as counts per cubic meter of air per hour. From these data, the pollen and mold calendars were constructed and expressed as counts per cubic meter of air per day. Skin prick tests were performed on more than 1000 patients attending the Pakistan Allergy, Asthma & Clinical Immunology Centre of Islamabad. The results indicated that there were 2 main pollen plants that contributed to seasonal allergies. These were *Broussonetia papyrifera *and *Cannabis sativa *during the March/April season and the July/September season, respectively. Although mold spores were continuously detected throughout the year, the most prominent mold was undetected mold and unconfirmed mold species similar to *Stachybotrys *species, which was high from July to September/October. Two additional molds contributing to allergic reactions were *Pithomyces *species and *Cladosporium *species, which were active during January and April, with the latter also being detected between October and November. These results may prove beneficial to both patients and physicians in planning a therapeutic protocol for avoidance and amelioration.

## 

Islamabad Federal Capital lies between 33° 28' and 33° 48' N and 72° 48' and 73° 22' E. It is bounded by Haripur district of the Khyber Pakhtunkhwa Province to the north and by Rawalpindi district of Punjab on all other sides. Islamabad is located on the northern most edge of the tract known as Pothohar plateau. Its population is 204, 000, with an area of 25 square miles (65 km^2^). "The topography is uneven containing several hills and valleys with elevations gradually rising from 500 to more than 1000 meters above sea level at the Margalla Hills."

There are 2 main flowering seasons in Pakistan, [1] that is, spring and fall. In Islamabad, [2] spring season is from February to April and second flowering period during autumn or fall season from July to September. The coldest month is January, when the mean maximum temperature is 30°C and the mean minimum temperature is 2.6°C. June is the hottest month with the mean maximum temperature of 44°C. The area has true distinct rainfall seasons. The summer season is from July to September during which monsoon precipitation (267 and 309 mm average for July and August, respectively) occurs. And the winter season is from December to April. The dominant flora on the top of the Margalla Hills include *Pinus roxburghii *(Cheel), *Acacia modesta *(Phulai), *Acacia arabica *(Kikar), *Olea ferrugenia *(Kohau), *Dodonaea *(Sanatha), *Justicia adhatoda *(Baker), *Carissa opaca *(Garanda), *Woodfordia fruticosa *(Dhabi), *Morus alba *(Tout), *Ficus carica *(Fig), *Ficus religiosa *(Peepal), and *Ficus benghalensis *(Bar). *Broussonetia papyrifera *(Paper Mulberry) is found in the foothills and plains of Islamabad.

### Aerobiology

Aerobiology deals with airborne microbodies, their dispersion, deposition, and implication on animals, human beings, and plants. These may be biological like pollen, dust, mold, bacteria, or organic particles and inorganic elements, including various types of gases, pesticides, smoke, etc., causing environmental pollution [International Association of Aerobiology (IAA)].

Tammeling[3] calculated that a person with normal activities inhales 8000 to 10, 000 L of air during 24 hours. This air proved often to be carrier of pathogenic materials causing allergies and infections. Aerobiological techniques, therefore, are closely related to clinical problems because continuous exposure of airborne pollen particulate affects the human system. Pollen and mold spores are involved in causing allergic rhinitis, asthma, and other allergic diseases in the human beings, [4] while Gupta and Chanda[5] described aerobiology in relation to respiratory allergy. Dry pollen can retain its capacity to elicit allergy indefinitely; however, pollen grains are ubiquitous as fossils and are probably more widely distributed in time and space than any other representatives of living matter [6]. This condition arises directly from the remarkable resistance, both to decay and to chemical decomposition of the substance known as sporopollenin, which is present in the walls of most pollen grains. Pollen and mold spores vary in size from 2 to 60 μm and contain protein allergenic constituents with a molecular weight from 10, 000 to 40, 000 Da [7].

Erdtman[8] presents data over a 50-year period. The average production of pollens per tree per year would be about the following, displayed in Table1

**Table 1:** Average production of pollens per tree per year

*Fagus sylvaticus *	409, 000, 000
*Betula alba *	5, 562, 400, 000
*Alnus *species	7, 269, 300, 000

Exact number of pollens produced by the *B. papyrifera *(paper mulberry) plant is not available but it falls in the category of trees producing catkins like *Alnus *species and *Betula *species, so each plant may be producing approximately 410 million to 7000 million pollens in a season.

## Materials and methods

The study materials consisted of 7-day recording volumetric spore trap (Burkard spore trap) provided by World Allergy Organization, Melinex tape, double-sided adhesive tape, transparent ruler (cutting block), scissors, brush, forceps, slides, gelvatol with basic fuchsine, and light microscope with ×400 magnification.

The study was conducted for 3 years from January 2005 to December 2007. The spore trap was placed at the height of 11 m and was allowed to run continuously. Airflow and other parameters were adjusted according to recommendations [9]. Every week, the collecting drum was prepared with Melinex tape fastened around the drum with double-sided adhesive tape joining both ends of Melinex tape, which is then coated with a thin layer of silicone grease.

The drum was removed on every Sunday at 9:00 AM and replaced with another drum coated as above at the same time. Permanent slides were prepared and particles (pollens and mold spores) counted according to recommended protocol. Pollen and mold spores were counted with ×400 magnification and recorded as counts per cubic meter of air per hour [9]. From the data of 3 years (2005, 2006, and 2007), pollen and mold calendars were prepared per cubic meter of air per day (counts per 24 hours according to protocol of IAA). But since the data were huge and we could not express them in this article, we rounded off and came up with average counts per cubic meter of air per hour per month. We have all the raw data and that can be presented any time if required.

Skin prick tests (SPTs) were done with the extracts purchased from Hollister-Steir (Spokane, WA). SPTs were done for pollens and molds and house dust and results were recorded (Table 7). SPTs and intradermal tests are the popular tests, [10,11] but SPT is more popular among allergists. Although direct comparisons indicate that intradermal test is more reproducible than SPT, there are many factors that favor the routine use of SPT. These include economy of time, patient comfort, and patient safety; the most important consideration is that results of SPT correlate better with clinical allergy.

## Results

Pollens and mold spores trapped by the Burkard spore trap were collected for 3 years (2005, 2006, and 2007). A total of 600, 240 pollens and 491, 904 fungal spores were trapped in 2005; in 2006, there were 560, 712 pollens and 546, 072 mold spores trapped; and in 2007, 559, 248 pollens and 433, 549 mold spores were trapped (Table 2). For convenience sake, the counts were divided into 4 groups (trees, grasses, weeds, and fungal spores). The occurrence of tree pollen during the year 2005 to 2007 (Table 3) dominated as compared with grass and weed pollen. A similar situation of a high pollen frequency was observed from subtropical Eastern Himalayas, Kurseong, and India [5].

**Table 2:** Total Pollen and Mold Counts in 2005, 2006, and 2007

	Pollens	Molds
2005	600, 240	491, 904
2006	560, 712	546, 072
2007	559, 248	433, 549

**Table 3:** Pollen Percentage Trapped in 2005, 2006, and 2007

**No**.		2005	2006	2007
1	Trees, %	81.98	82.64	80.7
2	Weeds, %	13.49	12.16	15.2
3	Grasses, %	03.17	03.65	2.35
4	Unidentified pollens, %	01.36	01.55	1.75

All the counts were originally counts per cubic meter of air per 24 hours according to protocol of IAA. Pollen calendar for each month was constructed, but it was huge data, which could not be adjusted in this article. These counts were rounded off to counts per cubic meter of air per hour in a month due to limitations of paper.

Pollen and mold calendar was prepared for 3 years with average counts per cubic meter of air per hour in a month (Tables 4, 5, 6). The data collected originally were counts per cubic meter of air per 24 hours according to protocol of IAA, and pollen calendar for each month was constructed, but the data were huge, which could not be adjusted in this article. These counts were rounded off to counts per cubic meter of air per hour in a month for convenience.

**Table 4:** Pollen and Mold Calendar 2005, per Cubic Meter of Air per Hour, January to December

Pollen Calendar 2005												
	**January**	**February**	**March**	**April**	**May**	**June**	**July**	**August**	**September**	**October**	**November**	**December**

Grass	1	7	5	4	1	1	1	1	2	1	1	1
*Morus alba *	1	2	16	5	1	1	1	1	1	1	1	1
*Broussonetia papyrifera *	1	3	490	72	6	4	7	4	9	1	1	1
*Pinus roxburghii *	1	2	1	1	1	1	1	1	1	0	0	0
Unidentified	1	1	1	1	1	1	1	1	1	1	1	1
*Cannabis sativa *	0	0	1	1	1	2	7	37	14	2	1	1
*Parthenium hysterophorus *	1	0	0	1	1	1	1	1	1	1	0	1
*Rumex chalepensis *	1	1	0	1	4	1	0	0	0	0	0	1
*Dodonaea viscosa *	1	1	1	5	1	1	0	0	1	2	0	1
*Eucalyptus *species	1	1	1	1	6	1	1	0	1	1	1	1
*Schinus molle *	1	2	1	0	0	0	0	0	0	0	0	0
*Cupressus *species	0	2	1	1	1	1	0	0	2	0	1	1
*Artemisia *species	0	0	0	0	0	0	0	1	1	2	1	1
*Chenopodium album *	0	0	0	0	0	0	0	1	2	1	1	1

**Mold Calendar 2005**												

	**January**	**February**	**March**	**April**	**May**	**June**	**July**	**August**	**September**	**October**	**November**	**December**

*Pithomyces *species	14	14	21	18	6	2	2	7	5	1	1	2
*Alternaria *species	3	1	4	7	6	4	4	3	9	3	1	2
*Cladosporium *species	11	12	25	37	9	4	6	6	6	13	17	8
Myxomycetes species	2	2	2	5	1	1	1	4	6	3	1	2
*Periconia *species	3	2	3	5	2	1	1	1	2	1	1	1
*Drechslera *species	1	1	1	1	1	1	1	5	6	1	1	1
*Aspergillus *species	1	2	2	1	1	2	1	1	1	1	1	1
*Leptosphaeria *species	1	1	1	2	1	1	2	3	1	1	1	1
*Trichia *species	0	0	1	1	0	0	1	1	1	1	1	1
*Curvularia *species	0	0	0	0	0	0	0	4	0	5	1	0
Unidentified molds	1	1	1	1	14	12	60	121	70	26	17	21

**Table 5:** Pollen and Mold Calendar 2006, Counts per Cubic Meter of Air per Hour, January to December

Pollen Calendar 2006												
	**January**	**February**	**March**	**April**	**May**	**June**	**July**	**August**	**September**	**October**	**November**	**December**

Grass	1	10	5	2	1	1	1	1	2	2	1	1
*Morus alba *	1	2	12	3	2	1	1	1	0	1	0	0
*Broussonetia papyrifera *	1	49	435	49	7	6	10	10	6	1	1	0
*Pinus roxburghii*	1	1	1	1	1	1	1	0	1	1	0	1
*Unidentified*	1	1	1	1	1	1	1	1	1	1	1	1
*Cannabis sativa *	1	0	1	1	2	2	9	12	14	2	1	1
*Parthenium hysterophorus *	1	1	1	1	1	1	1	1	1	1	0	1
*Rumex chalepensis*	1	1	1	1	4	1	0	1	0	0	0	0
*Dodonaea viscosa*	1	0	7	1	1	1	0	0	0	1	0	0
*Eucalyptus *species	1	1	1	1	6	1	0	0	1	1	1	1
*Cupressus *species	0	1	1	0	1	1	0	0	0	1	1	1
*Artemisia *species	0	0	0	0	0	0	0	1	1	3	1	1
*Chenopodium album*	0	0	0	0	0	0	0	1	1	2	1	1
*Schinus molle*	0	1	1	0	0	0	0	0	0	0	0	0
*Typha angustifolia *	0	0	0	0	0	0	0	1	0	0	0	0

**Mold Calendar 2006**												

	**January**	**February**	**March**	**April**	**May**	**June**	**July**	**August**	**September**	**October**	**November**	**December**

*Pithomyces *species	3	2	10	4	6	2	2	3	2	1	1	1
*Alternaria *species	14	14	21	18	6	2	2	7	5	1	1	2
*Cladosporium *species	15	9	24	13	8	3	4	5	5	18	19	7
*Myxomycetes *species	1	2	1	1	2	1	1	4	5	4	2	2
*Periconia *species	2	2	2	1	2	1	1	1	1	1	1	1
*Drechslera *species	1	1	2	1	1	2	1	5	4	2	1	1
*Aspergillus *species	2	2	2	2	2	4	1	2	4	1	1	1
*Leptosphaeria *species	1	1	1	1	1	1	2	5	2	1	1	1
*Trichia *species	0	0	0	0	0	0	1	1	1	1	1	1
*Curvularia *species	0	0	0	0	0	0	0	5	8	6	1	1
Unidentified molds	9	9	10	16	16	13	68	122	60	35	16	6

**Table 6:** Pollen and Mold Calendar 2007, Counts per Cubic Meter of Air per Hour, January to December

Pollen Calendar 2007												
	**January**	**February**	**March**	**April**	**May**	**June**	**July**	**August**	**September**	**October**	**November**	**December**

Grass	1	5	2	2	1	1	1	1	1	1	1	1
*Morus alba *	1	1	10	2	1	1	1	1	1	1	1	0
*Broussonetia papyrifera *	1	1	479	61	3	2	5	6	2	1	1	0
*Pinus roxburghii*	0	1	1	1	1	0	0	1	1	1	0	1
*Unidentified*	1	1	0	1	1	0	1	1	1	1	1	1
*Cannabis sativa*	0	1	1	1	2	8	26	23	7	1	1	1
*Parthenium hysterophorus *	0	0	0	1	1	1	1	1	1	1	1	1
*Dock sorrel*	0	0	0	1	1	1	0	0	1	1	1	0
*Dodonaea viscosa *	1	1	2	3	1	1	1	1	1	1	1	0
*Eucalyptus *species	1	1	1	1	2	0	0	0	1	1	1	1
*Cupressus *species	1	1	1	1	1	1	0	1	1	1	1	1
*Artemisia *species	0	0	0	0	0	0	1	1	1	1	1	1
*Chenopodium album *	0	1	0	1	1	0	1	0	1	1	1	1
*Acacia *species	0	0	0	1	1	0	0	0	0	1	0	0
*Schinus molle*	1	1	1	1	1	0	0	0	0	0	0	0
*Dandlion *species	0	0	0	0	0	0	0	0	0	1	0	0
TOTAL COUNT	8	15	498	78	18	16	38	37	20	15	12	9

**Mold Calendar 2007**												

	**January**	**February**	**March**	**April**	**May**	**June**	**July**	**August**	**September**	**October**	**November**	**December**

*Pithomyces *species	2	2	2	5	8	9	9	5	3	2	1	1
*Alternaria *species	1	1	1	2	2	1	1	2	2	2	2	1
*Cladosporium *species	5	13	8	14	13	8	5	5		11	6	7
*Myxomatous species *	2	1	1	1	1	1	1	3	2	1	1	2
*Periconia *species	1	2	1	3	3	1	1	1	1	2	1	1
*Drechslera *species	1	1	1	1	1	1	1	3	3	1	1	1
*Aspergillus *species	1	1	1	1	1	1	1	1	1	1	1	1
*Leptospira *species	1	1	1	1	1	1	2	2	1	1	1	1
*Trichia *species	1	1	1	1	1	1	1	1	1	1	1	1
*Curvularia *species	1	1	1	1	1	1	3	5	5	2	1	1
Unidentified molds	9	7	10	21	16	23	111	76	24	7	9	9

Tree, grass, and weed pollens and mold counts were graded into low, moderate, high, and very high per cubic meter of air per day according to recommendations [12-14].

Tree pollen grade is low if count is 1 to 14/m^3 ^air/d, moderate 15 to 89, high with 90 to 499, and very high > 500.

Weed pollen counts are low when count is 1 to 9/m^3 ^air/d, moderate 10 to 49, high are 50 to 499, and very high > 500.

Grass pollen are low when count is 1 to 4/m^3 ^air/d, moderate 5 to 19, high 20 to 199, and very high > 200/m^3 ^air/d.

Mold spores are low if count is 1 to 6499, moderate 6500 to 12, 999, high 13, 000 to 49, 999, and very high > 50, 000/m^3 ^air.

**Figure 1:**
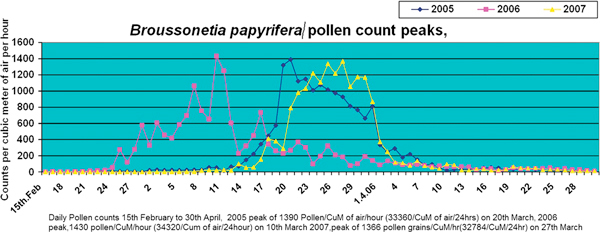
**Daily graph of *Broussonetia papyrifera *from 15th February to 30th April for 2005, 2006, and 2007**.

**Figure 2:**
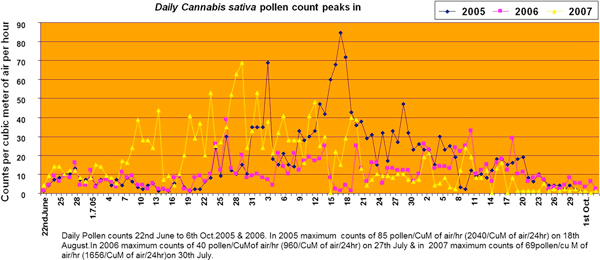
**Daily analysis of the frequency counts of *Cannabis sativa *between 22nd June and 6th October for 2005, 2006, and 2007**.

In our study, we graded the pollen and molds of allergenic importance like *B. papyrifera *(tree), *Cannabis sativa *(weed), mixed grass pollens, and mold. Graphs for main allergic pollen, that is, *B. papyrifera *and *C. sativa*, showing maximum pollen counts (peaks) are shown in Figures 1 and 2, respectively. There were large numbers of unidentified molds all the year round in 3 years.

The predominant pollen in 2005 was *B. papyrifera *during March and April, and *C. sativa *during August and September, whereas other pollens were those of grass and *M. alba*. The most plentiful molds during the January to April period were *Pithomyces *species and *Cladosporium *species.

In 2006, the pollen results were replicated with the addition of grass for February and *M. alba *for March. *Cannabis sativa *is most plentiful for August and September. There was a slight increase in *B. papyrifera *for July and August. During 2006, the most plentiful molds, as in 2005, were *Pithomyces *species (March) and *Cladosporium *species (January, March, April, October, and November).

In 2007, there was again replication of the above pollen count periods except *C. sativa *pollens, which were prevalent in July and August.

*Broussonetia papyrifera *belongs to the family Moraceae and has catkins, which contain about 200 flowers/catkin; therefore, each catkin may contain as many as about 3 to 6 million pollens. Moraceae pollen is approximately 14 to 15 μm in size and is an important cause of asthma and allergic rhinitis in susceptible individuals. Percentage of the pollen and molds per cubic meter of air is expressed in Table 3 and following were the results.

### Tree Pollen

The main flowering trees producing allergenic pollen in Islamabad were *B. papyrifera, Acacia *species, *Eucalyptus *species, *Pinus *species, *M. alba*, and *Schinus molle *(Tables 4, 5, 6).

2005: Total tree pollens trapped in spring were 81.98% (*B. papyrifera *71.3%, *M. alba *3.99%, *Pinus *species 1.21%, *Eucalyptus *species 1.98%, *Cupressus *species 1.21%, and others 2.9%).

2006: Tree pollens collected were 82.64% (*B. papyrifera *75.06%, *M. alba *3.14%, *Pinus *species 1.32%, *Eucalyptus *species 0.8%, and *Cupressus *species 0.3%).

2007: Tree pollens collected were 80.85% (*B. papyrifera *74.4%, *M. alba *2.70%, *Pinus *species 1.05%, and *Eucalyptus *species 1.95%, *Cupressus *species 0.91%, and others 0.26%).

#### Broussonetia papyrifera

*Broussonetia papyrifera *has huge vegetation in Islamabad and is present in almost every sector. Pollen trapped mainly in spring from end February to end April in 2005, 2006, and 2007 (Figure 1). Peak counts are variable depending on climatic factors in March [3].

In 2005, the count was 81.98% of total pollen count with maximum count (peak) of 1390 pollens/m^3 ^air/h on March 20th. Count was low (1-14 pollens/m^3 ^air/h) from January 24th to February 11th, moderate (15-89 pollens/m^3 ^air/h) from February 12th to 15th, high (90-499 pollens/m^3 ^air/h) from February 16th to 24th, and very high (.1500 pollens/m^3 ^air/h) from February 25th to April 22nd, reaching peak of 33, 360 pollens/m^3 ^air/d (or 1390 pollens/m^3 ^air/h) on March 20th. From April 23rd, the count started decreasing.

In 2006, count was 82.64% with maximum counts (peak) of 1430 pollens/m^3 ^air/h on March 10th (Figure 1). Pollens were first detected on February 11th and it was in moderate category from February 12th to 18th. Count was high from February 19th to 29th and very high from February 26th to April 12th, reaching peak of 34, 320 pollens/m^3 ^air/d (or 1430 pollens/m^3 ^air/h) on March 10th. After April 15th, it started decreasing till the end of October.

During 2007, the count was 80.85% of total pollen count with maximum count of 1366 pollens/m^3 ^air/h on March 27th. Pollens were detected on January 20th and low count continued till February 24th; it was in moderate category from February 25th to March 12th, and very high count from March 13th to April 11th, with peak count of 32, 784 pollens/m^3 ^air/h (or 1366 pollens/m^3 ^air/d). Pollen count was high from February 19th to 29th and very high from February 26th to April 12th, reaching peak of 34320 pollens/m^3 ^air/d (or 1430 pollens/m^3 ^air/h) on March 10th. During this period, there were no much fluctuations, and the count remained very high during this period. It was high from April 13th to May 11th and then decreasing with fluctuations till September 23rd. Climatic factors affected the different timings of peaks in 2005 and 2006.

### Weed Pollen

The pollen grains of weeds were recorded from mid-June to December (eg, *C. sativa, Artemisia *species, *Taraxacum *species, *Chenopodium *species, and *Madicago *species).

#### Cannabis sativa

*Cannabis sativa *is present in all the sectors of Islamabad, and pollens are the main cause of seasonal allergy in fall season. These pollen grains were trapped from March to November in 2005 and 2006 (Figure 2) but higher concentration from June 22nd to October 4th. The pollens of *C. sativa *are prevalent during fall season and cause of asthma, allergic rhinitis, and other allergic diseases during this season.

In 2005, maximum counts of 85 pollens/m^3 ^air/h were on August 18th (Figure 2). The 2005 series yielded a bimodal distribution, with peak counts occurring on August 4th and 18th with 70 and 85 counts/m^3 ^air/h, respectively. Pollen started appearing in March 11th and remained low to moderate till June 20th. It was high from June 21st to July 24th and very high from July 25th to September 8th, reaching peak of 2040 pollens/m^3 ^air on August 18th. From September 8th, the count started decreasing and was high from September 9th to October 11th, moderate from October 12th to 30th, and then it was absent in November.

In 2006, maximum counts of 40 pollens/m^3 ^air/h were on August 27th (Figure 2). Pollen started appearing from March 14th and were low till April 18th, moderate from April 19th to June 20, rising to high levels from May 8th to 15th high from June 21st July 24th, very high from July 25th to September 12th, with peak of 960 on July 27th. Then the count started falling, and it was high from September 13th to October 11th, moderate from October 12th to 29th, and then it was low/absent till end November.

In 2007, maximum counts of 63 pollens/m^3 ^air/h on August 30th (Figure 2). In 2007, pollen started appearing from March 3rd and till March 19th it was low. From March 20th to May 24th it was moderate. It was high from May 25th to July 8th and very high from July 9th to August 21st with peak of 1656 on July 30th, and then it started decreasing and was high from August 22nd to September 20th, moderate from September 21st to October 30th, and then it was low in November and absent by November 25th.

### Grass Pollen

The grass pollen grains could not be distinguished morphologically at generic or specific level. Pollen grains of the grasses from Islamabad were trapped all the year around and cause allergic symptoms. The common grasses of Islamabad include *Imperata cylindrica, Dichanthium annulatum, Bromus *species, *Chrysopogon aucheri, Heteropogon contortus, Phalaris minor *(Timothy grass), *Polypogon *species, *Sorghum halepense, Vitiveria *species, *Themeda anathera, Apluda mutica, Avena sativa*, and *Cynodon dactylon*, whose pollen grains have not been identified at the generic and specific level.

In Islamabad, our survey with Quaid-e-Azam University observed that there are 16 different grasses as mentioned in the results. We found the grass pollens in our study but could not study till the species level, so we named as mixed grass pollens. In 2005, pollens started appearing from January 13th and was low from January 13th to 31st, high from February 1st to 15th, very high from February 16th to March 9th, with peak of 956/m^3 ^air, moderate from February 14th to November 1st, and low to absent from November 1st to December 31st. So grass pollen was perineal with higher counts from February 1st to March 9th.

In 2006, grass pollen appeared on January 16th, low from 17th till January 31st, absent to moderate in January, high from February 1st to 15th, very high from February 16th to March 8th, with peak of 1187 pollens on February 17th, high to moderate from March 9th to November 12th, moderate to low from November 13th to December 13, and then it was absent till December 31st.

In 2007, pollen appeared on January 21st and was low to moderate till January 27th and high from January 28th to February 3rd, with peak of 315 pollens (very high) on February 4th. Count started falling and was high from February 5th to May 21st, moderate to high till October 2nd, moderate to low till December 6th, and then it was absent till end of year.

### Mold Spores

The molds are counted at ×400, and at this magnification, there are chances of missing good percentage of small *Cladosporium *spores, *Pithomyces *species, and *Alternaria *species. Mold spores are found throughout the year with periodic variation. *Pithomyces *species, *Alternaria *species, *Cladosporium *species, unidentified molds, *Drechslera *species, *Aspergillus *species, and *Curvularia *species were detected in our study. In our study, 10% patients coming to allergy center had SPT positive to molds like *Alternaria *species, *Cladosporium *species, and *Aspergillus *species. *Pithomyces *species, *Alternaria *species, *Cladosporium *species, *Drechslera *species, *Aspergillus *species, and *Curvularia *species have allergenic importance [14,16-19]. Unidentified spores were persistently present throughout the year reaching low category, and the spore counts were above 1000/m^3 ^in some days during the period of study, whereas other molds were having lower to absent spore counts in these years. In 2005 and 2006, the *Cladosporium *spores were above 1000 from March 25th to 31st, while it was in this range from February 18th to February 25th in 2007.

Table 7 presents the results of the SPTs for pollen, dust, and mold in patients suffering from asthma or allergic rhinitis in Allergy & Asthma Centre, Islamabad. Most of the patients were multisensitized. Of 1000 individuals from Islamabad, 875 (87.5%) had a positive reaction defined as a wheal size > 2 mm and 125 (12.5%) patients had negative SPTs. We had done a total of 14 SPTs with 12 different allergens and 2 controls (positive and negative). Most patients had SPTs positive to more than one allergens. Our result shows that most patients who were positive to pollen and mold also had positive response to house dust mite allergen. Maximum number of patients had positive test to house dust [446 (44.6%)], 419 (41.9%) had a positive SPT to *B. papyrifera*, and 267 (26.7%) to grass pollens. Weed pollens SPT results indicated that 220 (22%) were positive to *Cannabis*, 150 (15%) to burweed, 148 (14.8%) positive to dandelion, and 140 (14%) to pigweed. The results of SPT to molds were as follows: 161 patients (16.1%) gave positive reaction to mold mix, 135 (13.5%) to *Cladosporium*, and 10 (1%) to *Aspergillus *extract.

**Table 7:** Number and Percentage of Positive Reactions to the SPTs for the Specific Pollen, Mold, and House Dust Among the Patients Tested in This Study

Allergen	SPT Positive, n (%)
Total no. patients	1000
Positive	875 (87.5)
Negative	125 (12.5)
House dust	446 (44.6)
Paper mulberry *(Broussonetia papyrifera)*	419 (41.9)
Grass mix	267 (26.7)
*Cannabis sativa *	220 (22)
Mold mix	161 (16.1)
Dandelion *(Taraxacum officinale)*	148 (14.8)
Burweed	150 (15)
Pigweed	140 (14)
*Morus alba *	70 (7.0)
*Cladosporium (Hormodendrum)*	135 (13.5)
*Lambs quarter *	70 (7)
*Aspergillus *species	10 (1)

Most individuals were multisensitized.

## Discussion

Three years' data of aerobiology of Islamabad (2005, 2006, and 2007) indicate that Islamabad is among the cities with highest pollen counts in the world. In our study of 3 years, highest pollen count was that of *B. papyrifera *on 10th March 2006 (34, 320 pollens/m^3 ^air). The highest pollen count was seen in study of Domínguez[15] with data of last 25 years indicating that in Andalusia, South Spain, the higher pollen count belonged to *Olea europaea*. During only one day (May 22, 1991), 38, 393 olive pollen grains per cubic meter were detected in the air in Córdoba city [15].

For the first time, pollen and mold calendar of Islamabad was prepared from 3-year study (Tables 4, 5, 6). In spring (March to April), *B. papyrifera *pollens are the causes of severe allergic diseases like asthma, allergic rhinitis, and urticaria, whereas in fall (July to September), pollens of *Cannabis *are causing these allergic diseases. Grass pollens and molds are present throughout the year and are causing the above diseases in population of Islamabad.

It is not known that what concentrations of pollen and mold spores are required to cause asthmatic attacks, [14] but it is known that allergic effects are expected to vary with the individual mold sensitivity and fungal species. Results of several successful experimental trials of mold immunotherapy suggest that fungal antigens are clinically important [18].

There is possibility that fungal allergens may have more severe allergic effects in susceptible individuals compared with other allergens. For example, in Mayo study, [21] 11 asthmatic patients had one or more episodes of respiratory arrest during 1980 to 1989. Ten of 11 patients were skin test positive to *Alternaria *and had their respiratory arrest during the *Alternaria *aeroallergen season of summer and early fall. This view is supported by the study, which showed that greater magnitude of response per concentration of spores varied depending on the spore type [14].

*Parthenium hysterophorus *is a weed growing all over Islamabad. It has allergic importance[22] and is also a common cause of contact dermatitis in florists. Pollens are prevalent in July to September, but we did not capture much pollens with spore trap although it has thick vegetation in Islamabad. Size of pollen is about 18 μm.

Very little work has been done on aerobiology in Pakistan. Two published studies[1,2] were done on pollen counts, demonstrating that in Karachi and Islamabad there are 2 pollen seasons, that is, Spring and Fall, and the present study has confirmed this finding. Karachi University study found maximum total pollen count of 1378/m^3 ^air in one month (August 2001) with maximum count of grass pollens (816) in April (442 of Meliaceae pollen--tree pollen). In the present study, the maximum count of 34320/m^3 ^air was on one day (March 10, 2006). Maximum grass pollen in our study was 1187 pollen on 17th February, while it was 442 in one month (April 2001) in the Karachi study, so comparing the much lower counts of the tree and grass pollens in one month with our study, which shows much higher counts in a day.

Some differences were seen in the 2 studies; in the Karachi study, [1] researchers expressed the counts as pollen count per cubic meter of air per month, whereas we have selected units of pollen and mold count per cubic meter of air per day. We have extended our research beyond the family level to differentiate pollens at the species level as compared with their study at the family level. We also studied the mold counts per cubic meter of air per day, whereas Karachi group did not study the molds in the air. We also studied the SPTs with mold extracts, whereas Karachi group did not study the mold and SPTs.

This study showed that 2 main types of plants (ie, *B. papyrifera *and *C. sativa*) producing allergenic pollen and causing seasonal allergic diseases in Islamabad. Grass pollen and mold spore allergies are manifested perennially in susceptible individuals. These results are specific to the city of Islamabad, Pakistan. Further surveys need to be undertaken before generalization to a wider geographical region could be possible. These data are supported by another study in Chicago[22] from 1985 to 1989, which showed that death caused by asthma in 67 asthmatic patients aged 5 to 34 years was 2 times higher on days when fungal spore concentrations were at or above 1000 spores/m^3 ^versus the days when below 1000 spores/m^3^. In our study, the *Alternaria *spores are below 1000, whereas *Cladosporium *spores rise to above 1000 in spring season when the pollen count for *B. papyrifera *is very high, reaching to above 30, 000/m^3 ^air in a single day. There are deaths due to asthma in spring, and the pollen of *B. papyrifera *is regarded as the main cause, but above studies indicate that molds like *Alternaria *and *Cladosporium *may be the cause in aggravating asthma and even deaths. Research clearly shows that immunotherapy for the molds is effective, [18] so if the mold immunotherapy is included in the immunotherapy schedule in Islamabad, many complications and even deaths can be prevented.

## Conclusions

Within the limitations of this study, it may be concluded that the 2 main types of plants producing pollen and playing a role in the genesis of seasonal pollen allergies are *B. papyrifera *and *C. sativa *during March/April and July to September, respectively. Grass pollen and mold spores are manifested perennially in susceptible individuals. These results are specific to the city of Islamabad, Pakistan. Further surveys would have to be undertaken before generalization is possible to a wider geographical region.

## Competing interests

The authors declare that they have no competing interests.
